# Production of Potent Fully Human Polyclonal Antibodies against Ebola Zaire Virus in Transchromosomal Cattle

**DOI:** 10.1038/srep24897

**Published:** 2016-04-25

**Authors:** John M. Dye, Hua Wu, Jay W. Hooper, Surender Khurana, Ana I. Kuehne, Elizabeth M. Coyle, Ramon A. Ortiz, Sandra Fuentes, Andrew S. Herbert, Hana Golding, Russell A. Bakken, Jennifer M. Brannan, Steve A. Kwilas, Eddie J. Sullivan, Thomas C. Luke, Gale Smith, Gregory Glenn, Wenfang Li, Ling Ye, Chinglai Yang, Richard W. Compans, Ralph A. Tripp, Jin-an Jiao

**Affiliations:** 1 Virology Division, United States Army Medical Research Institute of Infectious Diseases, Fort Detrick, Maryland, USA; 2 SAB Biotherapeutics, Sioux Falls, South Dakota, United States of America; 3 Division of Viral Products, Center for Biologics Evaluation and Research (CBER), Food and Drug Administration, Silver Spring, Maryland, USA; 4 Viral and Rickettsial Diseases Department, Navy Medical Research Center, The Henry Jackson Foundation, Silver Spring, Maryland, USA; 5 Novavax Inc, Gaithersburg, Maryland, USA; 6 Department of Microbiology and Immunology and Emory Vaccine Center, Emory University, Atlanta, Georgia, 30322, USA; 7 The University of Georgia, Athens, Georgia, 30602, USA

## Abstract

Polyclonal antibodies, derived from humans or hyperimmunized animals, have been used prophylactically or therapeutically as countermeasures for a variety of infectious diseases. SAB Biotherapeutics has successfully developed a transchromosomic (Tc) bovine platform technology that can produce fully human immunoglobulins rapidly, and in substantial quantities, against a variety of disease targets. In this study, two Tc bovines expressing high levels of fully human IgG were hyperimmunized with a recombinant glycoprotein (GP) vaccine consisting of the 2014 Ebola virus (EBOV) Makona isolate. Serum collected from these hyperimmunized Tc bovines contained high titers of human IgG against EBOV GP as determined by GP specific ELISA, surface plasmon resonance (SPR), and virus neutralization assays. Fully human polyclonal antibodies against EBOV were purified and evaluated in a mouse challenge model using mouse adapted Ebola virus (maEBOV). Intraperitoneal administration of the purified anti-EBOV IgG (100 mg/kg) to BALB/c mice one day after lethal challenge with maEBOV resulted in 90% protection; whereas 100% of the control animals succumbed. The results show that hyperimmunization of Tc bovines with EBOV GP can elicit protective and potent neutralizing fully human IgG antibodies rapidly and in commercially viable quantities.

Ebolavirus (EBOV) belongs to *Ebolavirus* genus of the family *Filoviridae* and infects both humans and non-human primates (NHP) causing severe hemorrhagic fevers. Other symptoms of disease include sudden onset of fever, chills, headache, and anorexia followed by sore throat, vomiting, diarrhea, hemorrhaging, and the appearance of a petechial rash^1^,^2^,^3^. Filoviruses are categorized as Priority Class A pathogens by the Centers for Disease Control (CDC) and the National Institutes of Health (NIH); they present a clear biological warfare threat with mortality approaching 60–90% for certain viral subtypes^4^,^5^. The most recent outbreak of EBOV in Western Africa has clearly demonstrated that filoviruses pose a huge threat to public health worldwide. Presently, there are no licensed prophylactic or therapeutic countermeasures for EBOV infections in humans. Effective countermeasures that can be rapidly produced in clinically relevant quantities, such as vaccines, antivirals and other prophylactic and therapeutic treatments, are top research priorities.

In laboratory studies, treatment with multiple doses of KZ52, a human monoclonal antibody (Mab) derived from an EBOV survivor, prevented Ebola virus disease (EVD) in guinea pigs^6^; however, follow up studies in non-human primates (NHPs) failed to show measurable protection^7^. More recently, studies have demonstrated that purified macaque polyclonal IgG from convalescent monkey plasma, when given up to 48 hours post exposure, provides complete protection of NHP against filovirus challenge^8^. ZMapp (a cocktail of three humanized monoclonal antibodies produced in transgenic tobacco leaves) recently demonstrated a high level of protection in NHPs when given at 3 to 5 days after lethal challenge^9^,^10^,^11^. Convalescent plasma and ZMapp have been used in a small number of humans with EBOV infection, but logistical and production limitations have prevented widespread use^12^,^13^,^14^.

Current immunoglobulin products, such as human intravenous immunoglobulin (IVIG), monoclonal antibodies, and animal-derived polyclonal antibodies (pAbs), have known limitations. For example, human pAb products require a large volume of plasma, from many convalescent human donors with confirmed high titers, to make a commercial product^15^,^16^. Although animal-derived pAbs could be an alternative, they typically have very high reactogenicity as animal-derived antibody products are foreign proteins in humans. This can cause a variety of adverse effects, such as severe allergic reactions (anaphylaxis)^17^,^18^. To avoid serious side effects, animal antibodies are usually processed into smaller F(ab) or F(ab’)_2_ fragments, but this often reduces their half-life and potency. Animal derived monoclonal antibodies can be humanized or chimerized to human Fc fragments to avoid side effects, however, they are directed against a single epitope and may be subject to rapid mutational escape. This has led to the development of oligoclonal cocktails, but similar to monoclonal products, there are difficulties developing and producing enough of the oligoclonal product in a timely manner to assist in an outbreak scenario. It is clear that an innovative and rapid approach, combining the good safety profile of human polyclonal antibody products with the high neutralizing antibody activity derived from hyperimmune animals, is needed.

To address these limitations, SAB Biotherapeutics (SAB) has developed the Transchromosomic (Tc) bovine. The bovine immunoglobulin genes have been knocked out and a human artificial chromosome (HAC) containing the full germ line sequence of human immunoglobulin has been inserted, allowing the Tc bovines to produce fully human antibodies^19^,^20^,^21^,^22^. Like traditional animal systems used to produce polyclonal antibodies, Tc bovines can be hyperimmunized with vaccines containing strong adjuvants and/or immune stimulators, over an extensive period of time.

The Tc bovine system was previously used to produce anti-hantavirus polyclonal human IgG with high neutralization titers. This product was protective in two animal models of lethal hantavirus disease^23^. The therapeutic efficacy of anti-hantavirus human polyclonal antibody clearly demonstrated the proof-of-concept that it is possible to produce a candidate anti-viral biologic rapidly at high and scalable levels.

In this study, two Tc bovines were hyperimmunized with a vaccine containing nanoparticles comprised of recombinant full length EBOV/Makona GP. Fully human pAbs against EBOV, with high titers in a GP-specific ELISA, were generated and purified. The pAbs were then evaluated *in vitro* using virus neutralization assays and *in vivo* using a lethal mouse model challenged with maEBOV. It was shown that intraperitoneal (i.p.) administration of the purified fully human IgG (100 mg/kg) from Tc bovine plasma collected post 2^nd^ vaccination was sufficient to protect BALB/c mice from a lethal challenge with maEBOV. This data demonstrates that the Tc bovine system can be used as a countermeasure to rapidly, and in large quantities, produce fully human antibodies to treat against EBOV.

## Results

### Production of human antibodies in Tc bovines vaccinated with a vaccine containing recombinant EBOV GP

#### Immunization schedule and sample collection

Two Tc bovines (ID #2134 and #2136) were immunized with EBOV/Makona recombinant GP formulated with SAB’s proprietary adjuvant (SAB-adj-1), that has been demonstrated to produce high antibody titers in cattle^21^. A total of 8 immunizations were conducted at three to four week intervals. For the first 4 vaccinations, a dose of 2 mg EBOV-GP [administered by intramuscular (IM) injections] was used; subsequently a higher dose of 5 mg of EBOV-GP (administered IM) was used for the fifth through eighth immunization. Serum was collected from each Tc bovine prior to and 8–10 days post each vaccination (V), and was evaluated using ELISA titer assay and virus neutralization tests. Large volumes of plasma were collected from each Tc bovine prior to immunization and on days 8–14 post each vaccination between V2 and V8.

#### EBOV rGP specific antibody titer in serum and purified samples as determined by ELISA

**Figure 1:**
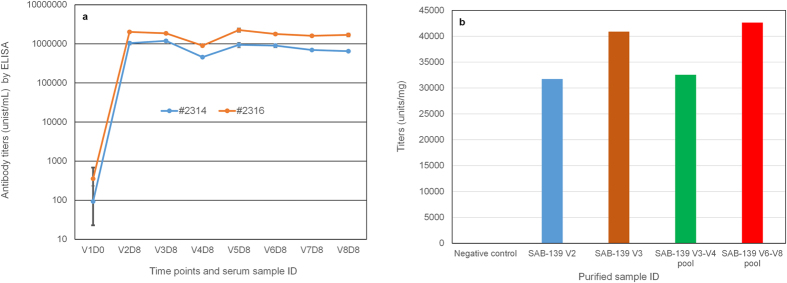
Anti- EBOV/Makona rGP titers by ELISA. (**a**) Sera collected from two hyperimmunized Tc bovines (#2314 and #2316) were measured for endpoint binding titers (units/mL) by rGP specific ELISA. Sera were collected 8 days post each vaccination from the second vaccination (V2) to eighth vaccination (V8). V1D0 represents the pre-immunized titers. Student’s T-test method was used for analysis of ELISA titers for V2D8 vs V1D0. (**b**) ELISA endpoint binding titers (units/mg) of purified fully human polyclonal antibody samples.

To evaluate the antigen-specific IgG response in Tc bovines following immunizations with the EBOV/Makona recombinant GP (rGP) vaccine, an EBOV/Makona rGP specific ELISA was performed with the serum samples (Fig. 1a). GP-specific ELISA titers increased significantly after the second vaccination in both Tc bovines (V2 vs V1, p = 1.32 × 10^−13^). Interestingly, the antibody titer was slightly increased in Tc bovine #2314 but decreased in Tc bovine #2326 following the third vaccination. Antibody titers continued to decline in both vaccinated Tc bovines at the fourth vaccination. Given this phenomenon, and to determine if GP-specific antibody titers could be boosted, GP vaccine containing 5 mg GP was administered from the fifth to eighth vaccinations. Using the 5 mg GP dose, it was observed that there was a trend towards increased ELISA titers that were maintained at high levels from V5 to V8. Negative control antibody was purified from pre-vaccination Tc bovine plasma. Fully human antibody against EBOV GP was purified from plasma collected from V2, V3, V3–V4 pooled plasma and V6–V8 pooled plasma, respectively. The purified fully human antibody against EBOV was designated as SAB-139. These representative purified anti-EBOV samples were also evaluated for ELISA titers (Fig. 1b). Titers were expressed as units/mL for serum samples (Fig. 1a) and units/mg of human IgG for purified samples (Fig. 1b). These results clearly show that the human antibody responses elicited in both Tc bovines specific to EBOV rGP are robust. Taken together, this data indicated that high titer human antibodies targeting EBOV GP can be generated by the hyperimmunized Tc bovines, and that purified human IgG retained high titer binding activity against EBOV rGP.

#### Virus neutralizing antibody activity in serum and purified samples

**Figure 2:**
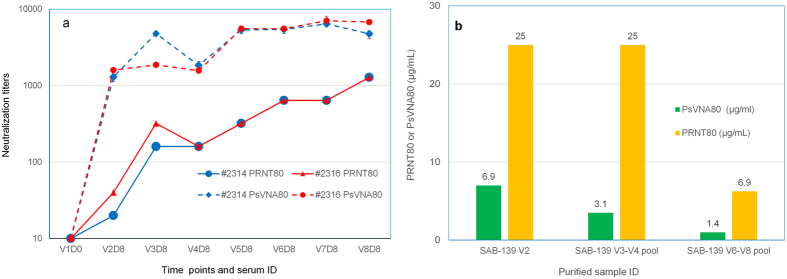
Virus neutralization titers or activities of anti-Ebola antibody in Tc bovine serum samples and purified fully human pAbs. (**a**) PRNT_80_ and PsVNA_80_ titers of V2–V8 serum samples. For PsVNA_80_ titers, symbols represent the mean ± SD of triplicates. For PRNT_80_ titers, each sample were run in duplicates and 80% neutralization of virus was observed at the same dilution factor, thus no error bars. The limit of quantitation was a titer of 20 for PsVNA_80_ and 10 for PRNT_80_. Samples with titers <20 or <10 were given a value of 10. (**b**) PRNT_80_ and PsVNA_80_ data of purified fully human pAbs. The PsVNA titer for the V2 pool is the mean for triplicates from a single assay. PsVNA titers for the V3–V4 and V6–V8 pools are the geometric mean of two independent experiments. Student’s T-test method was used for analysis of antibody neutralization titers.

To evaluate the neutralizing antibody responses generated in the hyperimmunized Tc bovines, a plaque reduction neutralization test (PRNT)^24^ against wild type EBOV and a pseudovirion neutralization assay (PsVNA)^25^ using vesicular stomatitis virus (VSV) pseudotyped with the GP protein of EBOV were performed using serum samples collected after immunizations from each Tc bovine. Figure 2a shows PRNT_80_ and PsVNA_80_ titers for serum samples collected from V2 to V8. PRNT_80_ and PsVNA_80_ titers refer to the highest serum dilutions required to achieve 80% viral inhibition. PsVNA_80_ titer increased significantly post V2 (V2 vs V1, p = 0.0016) and continued to increase up to V8 for both Tc bovines, except a slight decrease in V4 samples. PRNT_80_ titer increased significantly post V3 (V3 vs V1, p = 0.012 for #2314 and p = 0.004 for #2416) and continued to increase up to V8 for both Tc bovines, except a slight decrease in V4 samples. Representative purified fully anti-EBOV human IgG samples (V2, V3–V4 pool or V6–V8 pool purified samples were prepared from plasma pool of two Tc bovines) were also measured for virus neutralization activities by values of PRNT_80_ and PsVNA_80_ (Fig. 2b), presented as the lowest IgG concentration required to achieve 80% viral inhibition. Data indicated that purified fully anti-EBOV human IgG samples, from different time points or pooled plasma, retain potent neutralization activities against EBOV.

#### Hyperimmunization of Tc bovines with rGP vaccine promotes better antibody affinity maturation to native conformational rGP compared to denatured rGP

In the current study, rGP proteins were used in surface plasmon resonance (SPR) to capture the real-time kinetics of antibody association and dissociation rates, which reflects the overall antibody binding avidity. In previous studies, we observed that conformation-dependent GP-specific neutralizing monoclonal antibodies bind to the rGP proteins from EBOV and Sudan virus (SUDV) in SPR using His_6_-tag captured protein on HTG chips, confirming conservation of native GP structure. However, these Mabs lost binding to rGP protein that was amine-coupled directly to GLC chips, suggesting partial denaturation of the GP protein and loss of conformational epitopes (data not shown).

**Figure 3:**
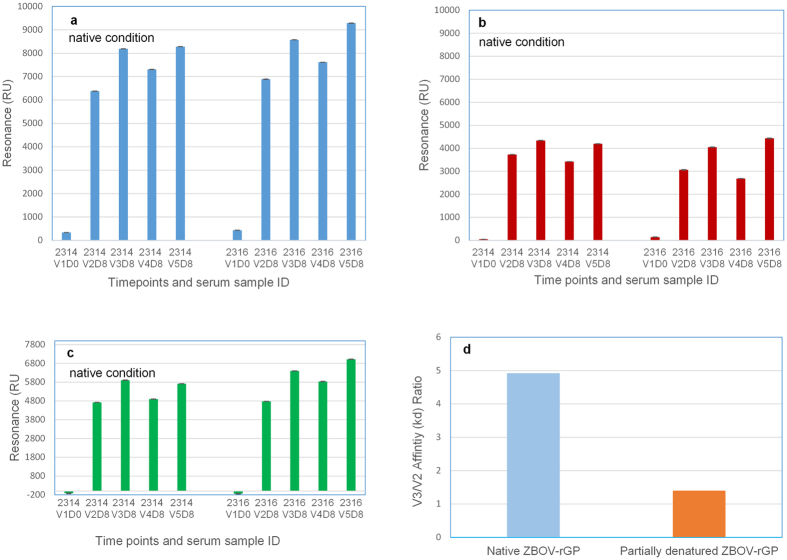
Affinity of anti-EBOV antibody for rGP proteins measured by SPR. Steady-state equilibrium analysis of the binding of Tc bovine IgG preparations to different rGP proteins was measured under native conditions (rGP proteins immobilized on a HTG sensor chip). (**a**) total binding of antibodies to EBOV-GP; (**b**) total binding of antibodies to SUDV-GP; and (**c**) total binding of antibodies to EBOV N-terminal half of GP (1–308 residues) containing receptor binding domain. Student’s T-test method was used for analysis of affinity data. (**d**) Increase in antibody affinity from V2 to V3 as measured by SPR. Antibody affinity (off-rate constants, kd) to EBOV-GP were determined under native and partially denatured conditions. V3/V2 affinity (kd) ratios of antibody binding to native (left column) or partially denatured rGP (right column) shown are calculated by dividing the kd of V3 by kd of V2 for antibody binding to native or partially denatured rGP, separately.

The negative control Ig serum sample (prior to immunization sample, V1D0) showed very low reactivity to rGP proteins in SPR. Binding of 1 mg/mL Ig post second vaccination (V2) to native homologous EBOV rGP increased strongly (V2 vs V1, p = 0.00076) following 2^nd^ vaccination, but the antibody reactivity peaked after the 3^rd^ vaccination (V3) (Fig. 3a). These SPR data differ from the ELISA results (Fig. 1a: wherein the ELISA binding peaked after 2^nd^ vaccination) and may reflect binding to more non-conformational linear epitopes in the ELISA. Importantly, the SPR results are in agreement with the neutralization assays. We also explored binding to the receptor binding domain (RBD) using a recombinant N-terminal half of GP (1–308 residues) containing RBD (Fig. 3b). The pattern of binding was identical to that of the binding to EBOV-GP, suggesting that significant fraction of the vaccination-induced antibodies from boosts V2–V5 were targeting epitopes mapping to the N-terminal half of GP containing RBD, which contains key protective targets. The EBOV rGP post-vaccination antibodies also showed cross-reactivity (although at lower Max RU) to the SUDV rGP in the native SPR assay at 1 mg/ml Ig purified from the V2–V5 immune sera (Fig. 3c). To further investigate if repeated vaccinations with rGP promote antibody affinity maturation and lead to an increase in antibody avidity, we determined the off-rate constants of V2 and V3 Tc bovine Ig samples following binding to the native EBOV rGP. We compared antibody binding off-rates to native rGP with the binding to partially denatured rGP in order to distinguish binding to conformational vs linear/denatured epitope specific antibodies as previously described^26^,^27^. Immune Ig obtained from Tc bovines demonstrated ~5-fold increase in binding affinity with slower dissociation rate constants (>10^−3^/sec) following V3 compared to V2 against the native EBOV rGP (Fig. 3d). That difference in affinity was significantly diminished when partially denatured rGP was used, confirming that the high affinity binding after V3 was primarily due to conformational-dependent epitope-specific antibodies.

#### Protective efficacy of purified fully human antibody against EBOV in mice

**Figure 4:**
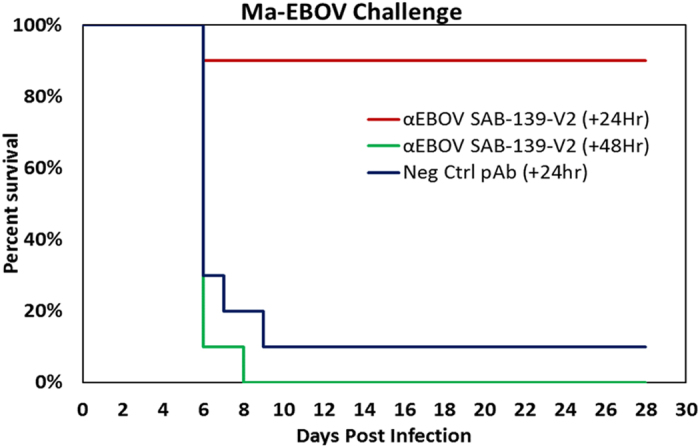
ma-EBOV mouse challenge study with purified fully human polyclonal antibody against Ebola (SAB-139-V2). BALB/c mice (n = 10/group) received a single intraperitoneal injection of 100 mg/kg of purified negative control pAbs or 100 mg/kg of SAB-139-V2 24 hr or 48 hr after lethal challenge with 100 PFU ma-EBOV by intraperitoneal route. Kaplan-Meier survival analysis is shown indicating the percentage of surviving mice for 28 days post challenge. A two tailed Fisher’s exact test was used to analyze mouse survival data on day 28.

To evaluate the protective efficacy of purified fully human antibody against EBOV, IgG was purified from plasma pools of two Tc bovines post 2^nd^ vaccination (designated as SAB-139-V2) and administrated to BALB/c mice that were challenged with a lethal dose of ma-EBOV. In this experiment, after 24 or 48 hours post challenge with 100 PFU ma-EBOV by the intraperitoneal (IP) route, BALB/c mice (n = 10/group) received a single IP injection of either 100 mg/kg of purified negative control pAbs or 100 mg/kg of SAB-139-V2. Kaplan-Meier survival analysis is shown indicating the percentage of surviving mice for 28 days post challenge (Fig. 4). Nine of 10 mice receiving negative control antibody, and 10 of 10 mice receiving SAB-139-V2 48 hour post challenge died. However, nine of the 10 mice treated with SAB-139-V2 24 hours post challenge survived the lethal EBOV infection. The mice that survived showed clinical signs of disease include ruffled fur and lethargy. Compared with the control mice, a significant protection was observed for the mice treated with SAB-139-V2 24 hr after challenge with 100 PFU ma-EBOV (p = 0.001).

## Discussion

Previous studies using a codon-optimized DNA vaccine expressing the EBOV GP gene showed that protective antibodies were generated and complete protection was observed in mice and monkeys challenged by EBOV^28^. Very recently, a vaccine containing recombinant EBOV/Makona GP nanoparticles was shown to be 100% protective in *Cynomolgus macaque* monkeys, and generated sustained IgG anti‐GP responses in baboons (Novavax, unpublished data). Here, we hyperimmunized two Tc bovines that expressed high levels of fully human IgG^22^ with a vaccine comprised of EBOV/Makona rGP nanoparticles and SAB’s proprietary adjuvant. Our goal was to validate the robust immune responses of EBOV/Makona rGP nanoparticle as observed in monkeys, and more importantly to produce large quantities of human pAbs that would be suitable for passive immune therapy in filovirus-infected humans. Both Tc bovines in this study developed high neutralization antibody responses as measured by PRNT and PsVNA after 3^rd^ vaccination. It is notable that a higher dose of recombinant EBOV/Makona GP nanoparticles administered at the 5^th^ vaccination further increased the level of neutralization activity and these increased neutralization antibody responses were maintained at high levels through the 8^th^ vaccination.

Currently, a combination of VSV-PsVNA and PRNT are the most commonly used assays to determine the neutralizing antibody responses to EBOV antigen/vaccine^25^. The results from both assays show a similar trend with virus neutralization titers increasing after each vaccination post V2, although the antibody neutralizing titer (less dilutions of serum or higher antibody concentrations) measured by PRNT_80_ is lower than that measured by PsVNA_80_. One possible reason for this difference is that the PsVNA measures GP-mediated particle entry, and not subsequent steps required for plaque formation (e.g., virion egress). Another possibility is that less GP-specific antibody is required to neutralize the VSV pseudovirion than EBOV virions due to virion size differences. The EBOV particles (~80 × 800 nm) are larger than VSV particles (~70 × 200 nm), and thus could potentially contain more GP trimers on their surface and would require a larger quantity of antibody to achieve neutralization. Similarly, the GP expression could be higher per surface unit area on EBOV than the pseudovirion, thus requiring more anti-GP antibody to achieve neutralization.

To evaluate the quality of antibody binding to native GP, SPR based real time kinetics was performed. This approach also allowed measurements of polyclonal serum antibody dissociation rates following each vaccination, which can be used as a surrogate of antibody affinity. Importantly the SPR allowed comparison of binding to conformationally intact vs. partially denatured rGP. After 3^rd^ vaccination about 5-fold increase in antibody affinity to native rGP was seen compared with post 2^nd^ vaccination Ig. However this increase in Ig affinity was not observed against partially denatured/linear epitopes within Ebola-GP (Fig. 3d). The SPR data was in very good agreement with the PRNT assay, demonstrating a significant increase in total binding and higher affinity towards the native rGP after the 3^rd^–5^th^ vaccinations. Approximately, 70% of anti-GP binding antibodies in the Tc bovine sera following vaccination recognize the N-terminal half of GP containing RBD suggested targeting of key protective epitopes on the virus, but remaining 30% of antibodies recognize other sites within native GP. The observed cross-reactivity against Sudan-GP and increase in antibody affinity following 3^rd^ vaccination in SPR suggest that this Ig may have therapeutic potential against more than one Ebola type and should be further evaluated in animal studies.

Rodent experimentation to determine clinical efficacy is a first step in the proof of concept to identify attractive candidates to move forward into NHP experiments that more closely model EVD in humans. Although Tc bovine-derived anti-EBOV antibody provided 90% protection when administered one day post lethal challenge; no protection was afforded when the antibody was given 2 days post challenge. The reason for no protection 2 days post challenge is perplexing; however, similar effects on the timing of treatment on the capacity of anti-EBOV monoclonal antibodies have been reported. For example, studies by others also have shown increased survival of rodents challenged with maEBOV when treatment with mouse Mabs was initiated at 1 or 2 days post infection^29^. In addition, based on the SPR antibody binding/affinity measurements and the PRNT results, it is highly likely that passive transfer of Tc Ig from later boosts (V3–V8) with higher antibody affinity could provide better protection, including at 48 hours post challenge. More studies in rodents and NHP are needed in order to establish the treatment window for the Tc bovine-derived fully human IgG.

In summary, this study provides a proof of concept demonstrating that filovirus GP vaccine can be used in a Tc bovine production system to produce a fully human polyclonal IgG product that offers post-exposure protection against filovirus infection. Others have previously demonstrated that individual Mabs and Mab cocktails can protect mice and guinea pigs from lethal challenge with filoviruses^6^,^28^; however, neither of these animal models is necessarily predictive of protection in the gold standard NHP model of EBOV disease^7^,^8^,^11^. Future preclinical studies, including NHP studies, will provide additional insight into the further development of this fully human anti-EBOV antibody product produced using Tc bovine platform technology.

The Tc bovine platform to produce human IgG is relatively new, and products have only recently advanced towards clinical trials. Nevertheless, there are several features of the Tc bovine platform that are pertinent to developers of medical countermeasures targeting newly emerging pathogens. Tc bovines can be immunized with as many known antigens as possible (e.g., a combination of different Ebola virus species such as *Zaire*, Sudan and Taï Forest) to produce hyperimmune plasma containing multi-valent polyclonal antibodies, which can then be purified to generate final product as stockpiles for future use. Tc bovines can also be used to manufacture relatively large quantities of new products rapidly (3–5 months) against any new emerging infectious disease as long as the antigen/vaccine (pathogen) known to be the target of protective antibody is readily available. Currently, the US Food and Drug Administration has provided a clear regulatory pathway to approve polyclonal antibody products derived from human plasma or animal plasma. There is reason to believe that the Tc bovine-derived polyclonal human IgG, including the anti-EBOV neutralizing and protective antibody described herein, could follow a similar regulatory pathway.

## Methods

### Antigen preparation

The antigen in this study was a recombinant full length EBOV/Makona glycoprotein (rGP) trimers expressed in insect cell lines that, when purified^30^, assembled into 30–45 nm particles formed from 2–9 rGP trimes (rGP nanoparticles).

### Transchromosomal bovines

All experimental protocols related to production of Tc bovines, immunization and sample collections described in this study were reviewed and approved by the Institutional Animal Care and Use Committee (IACUC) at SAB Biotherapeutics Inc. All experiments were performed in accordance with the approved guidelines for animal care and management of research projects.

Tc bovines were produced as previously described^19^,^20^,^21^,^22^. The Tc bovines used in this study are homozygous for triple knock-outs in the endogenous bovine immunoglobulin genes (*IGHM*^*−*/*−*^ *IGHML1*^*−*/*−*^ *IGL*^*−*/*−*^) and carry a human artificial chromosome (HAC) vector labeled as isKcHACD^22^. This HAC vector consists of human chromosome 14 fragment, which contains the entire human immunoglobulin heavy chain locus except that the IGHM constant region remains bovine and the key regulatory sequences were bovinized; and human chromosome 2 fragment, which contains the entire human immunoglobulin k light chain locus^19^,^20^,^21^,^22^. Tc bovines were produced by using genetically engineered and cryobanked fibroblast cells as chromatin donors via a proprietary chromatin transfer (CT) procedure.

### Tc bovine hyperimmunization and sample collection

Two Tc bovines were intramuscularly immunized with EBOV/Makona recombinant GP (2014 Zaire strain, from Novavax Inc.) formulated with SAB’s proprietary adjuvant SAB-adj-1. The Tc bovines were vaccinated 8 times (V1–V8) at three to four week intervals. The antigen dose was 2 mg per animal for V1 to V4, and 5 mg per animal for V5 to V8. Plasma and serum samples were taken at various time points before and/or after each vaccination. All protocols were approved by the SAB Biotherapeutics IACUC.

Prior to the first vaccination (V1), a volume of pre-vaccination plasma was collected from each study Tc bovine to be used as negative control. Up to 2.1% of body weight of hyperimmune plasma per animal per time point was collected from immunized Tc bovines for anti- EBOV fully human polyclonal antibody production [on days 8, 11 and 14 post each vaccination starting from the second vaccination (V2) to the eighth vaccination (V8)]. Plasma was collected using an automated plasmapheresis system (Baxter Healthcare, Autopheresis C Model 200). Plasmas were stored frozen at −20 °C until purifications were performed.

### Purification of fully human IgG

Fully human IgG negative control was purified from plasma collected from Tc bovines prior to the first vaccination. Plasma collected from each vaccinated Tc bovine [on days 8, 11 and 14 post each vaccination starting from the second vaccination (V2) to the eighth vaccination (V8)] was the source material for purification of fully human anti-EBOV pAbs. Fully human IgG purification was performed as previously described^22^,^23^. Briefly, frozen plasma is thawed overnight at 25 °C, pooled, and pH adjusted to 4.8 with 20% acetic acid. Then the pH adjusted plasma was fractionated with caprylic acid in combination with a filtration step using a depth filter to remove non-IgG proteins. The filtrate was then adjusted to pH 7.5 with 1 M Tris, further purified by using human IgG light chain kappa specific affinity chromatography, and followed by a second purification using bovine IgG heavy chain specific affinity chromatography. The purified fully human anti-EBOV IgG (SAB-139) is in a sterile liquid containing the following formulation buffer: 10 mM glutamic acid monosodium salt, 262 mM D-sorbitol, 0.05 mg/mL Tween80, pH 5.5. This formulation buffer was developed to keep IgG stable in liquid form over an extended period of time.

### ELISA titer

Determination of EBOV rGP-specific human IgG antibody endpoint titers were performed in 96-well HB ELISA plates coated overnight at 4 °C with 100 μL/well of 2 μg/mL recombinant EBOV/Makona GP (Novavax) in PBS. Plates were washed with PBST (PBS with 0.05% Tween 20) and blocked at room temperature (RT) for 1 hour with 1% BSA in PBS. After washing with PBST, serum samples or purified fully hIgG SAB-139 were added to the plates with serial dilution in PBST and incubated for 1 hr at RT. Following washing with PBST, goat anti-human IgG-Fc conjugated with horseradish peroxidase (HRP) (Bethyl) was added to plates and incubated for 1 hr at RT. After final washing with PBST, the bound anti-EBOV rGP antibodies were detected colorimetrically by using a TMB substrate kit. The absorbance was read in a microplate reader at 450 nm. The standardized serum is assigned an endpoint antibody titer value equivalent to the reciprocal dilution that produces a positive optical density of three times greater than the blank optical density. For serum ELISA titers, triplicates were done for each sample except V8D8 samples, which were performed as duplicates. Data were reported as the mean ± SD of triplicates (V2–V7) or duplicates (V8). Antibody titers are reported in units/mL for serum samples, and in units/mg for purified IgG samples. The titer value (units/ml) is defined as the highest dilution of serum sample where the OD450 reading was 3-fold higher than blank. The titer activity value (units/mg) is defined as the highest dilution of 1 mg of purified antibody where the OD450 reading was 3-fold higher than blank.

### Plaque Reduction Neutralization Test (PRNT)

PRNT was performed as previously described^24^. Serum samples collected from each vaccinated Tc bovine were heat inactivated at 56 °C for 30 min. An initial 1:5 dilution of the heat inactivated sera was made, followed by two-fold serial dilutions. Samples were diluted in complete Eagle’s minimum essential medium, with Earle’s salts containing 2% heat inactivated FBS and 0.05% Gentamicin, and analyzed in duplicate. An equal volume of complete Eagle’s minimum essential medium (cEMEM) with Earle’s salts, supplemented with 10% guinea pig complement (Cedarlane) containing 100 PFU of Zaire virus, was added to the sera dilutions and incubated at 37 °C for 1 hour. Following incubation, Vero cell monolayers were inoculated, overlaid with agarose and incubated at 37 °C. A second agarose overlay containing 5% neutral red was added 7 days later, with plaques (Zaire) counted the following day. Neutralization activity was determined to be the IgG concentration in serum or purified Ig samples that reduced the number of plaques by 80% compared with the control wells. For serum neutralization titers, duplicates were done for each sample. Data were reported as the mean ± SD of duplicates. Purified IgG samples were measured using the same methods as for serum samples, but without heat treatment.

### Pseudovirion Neutralization Assay (PsVNA)

PsVNA was performed as previously described^25^. Pseudovirions (PsV) that express luciferase were prepared in HEK 293T cells as previously described using the EBOV-GP_co_ plasmid to express the EBOV glycoprotein^31^. Sera samples collected from the vaccinated Tc bovines were heat inactivated at 56 °C for 30 min. Initial 1:10 dilutions of sera (in triplicate) were made, followed by five-fold serial dilutions in cEMEM with Earle’s salts containing 10% heat inactivated FBS, 10 mM HEPES (pH 7.4) and 100 IU/ml penicillin, and 100 μg/ml streptomycin. An equal volume of cEMEM supplemented with 10% human complement (Sigma) containing 10^5^ focus-forming units/mL of PsV was added to the serum dilutions and incubated overnight at 4 °C. Following incubation, Vero cell monolayers seeded in flat, clear bottom, black-walled 96-well plates (Corning) were inoculated with 50 μl of the PsV:Tc bovine serum mixture and incubated at 37 °C for 18–24 hours. The medium was discarded, the cells were lysed, and luciferase substrate was added according to the *Renilla* Luciferase Assay System protocol (Promega #E2820). The luciferase data was acquired using a Tecan M200 Pro microplate reader. The raw data was graphed using GraphPad Prism to calculate percent neutralization. The data was fit to a four-parameter logistic curve and the PsVNA_80_ neutralization activity was interpolated as the IgG concentration in serum or purified samples that inhibited 80% of luciferase activity. For serum neutralization titers, minimal two tests were done for each sample. Data was reported as the mean ± SD of triplicates. Purified IgG samples were not heat-inactivated before evaluation in the assay.

### Binding of Tc bovine Ig to recombinant GP proteins and off-rate measurements by surface plasmon resonance (SPR)

Steady-state equilibrium binding of pre- and post-GP vaccinated Tc bovine Ig was monitored at 25 °C using a ProteOn surface plasmon resonance (Bio Rad). Recombinant GP (1–650 residues) and rGP-N-terminal half containing RBD (1–308 residues) were expressed with a polyhistidine tag at the C-terminus, and purified using Ni-NTA chromatography. The Ebola-GP proteins were coupled to a GLC sensor chip with amine coupling at pH 4.5 (partial denaturing condition) with 100 resonance units (RU), or were captured on an HTG surface (native condition) via the His_6_ tag at 100 RU in the test flow cells. Samples of 200 μL freshly prepared Ig preparations at 1 mg/mL and 0.1 mg/mL were injected at a flow rate of 50 μL/min (240-sec contact time) for association, and dissociation was performed over a 1200 second interval (at a flow rate of 50 μL/min). Responses from the protein surface were corrected for the response from a mock surface and for responses from a separate, buffer only injection. Monoclonal antibody 2D7 (anti-CCR5) was used as a negative control in these experiments. Binding kinetics for the Ig preparations and data analyses were calculated with BioRad ProteOn manager software.

Antibody off-rate constants, which describe the stability of the complex, i.e. the fraction of complexes that decays per second, were determined directly from the Ig preparation sample interaction with rGP protein using SPR (as described above) and calculated using the BioRad ProteOn manager software for the heterogeneous sample model. For all polyclonal Ig, it was important to demonstrate that the dissociation rates were independent of total GP-binding antibody titers. To that end, parallel dissociation curves for 1 mg/ml and 0.1 mg/mL for each post-vaccination Ig sample were established as previously described^27^. To improve the measurements, the off-rate constants were determined from two independent SPR runs.

For SPR based affinity data, all samples were tested twice in duplicates, so data from a total of 4 replicates was used for the SPR analysis.

### Protective antibody activity in a virus challenge mouse model

Female BALB/c mice aged 6 to 8 weeks were used in all challenge experiments with mouse-adapted EBOV (maEBOV) as described by Bray *et al.*^32^. Briefly, mice were injected with 100 PFU of maEBOV diluted in PBS. Prior to use of maEBOV at 100 pfu, a dosing experiment was completed. Lethality was observed to be higher at 100 pfu than at the traditional 1000 pfu (95% and 85% respectively). Therefore all of our challenges in BALB/c mice are completed with the more lethal 100 pfu infection dose. All challenge studies involving the use of maEBOV were performed at USAMRIID in an animal Biosafety level 4 laboratory. All experimental protocols related to challenge mouse model described in this study were reviewed and approved by the Institutional Animal Care and Use Committee (IACUC) at United States Army Medical Research Institute of Infectious Diseases. Research was conducted under the approved IACUC protocol in compliance with the Animal Welfare Act, PHS Policy, and other Federal statutes and regulations relating to animals and experiments involving animals. The facility where this research was conducted is accredited by the Association for Assessment and Accreditation of Laboratory Animal Care, International and adheres to principles stated in the Guide for the Care and Use of Laboratory Animals, National Research Council, 2011.

Negative control pAbs and anti-EBOV pAbs were diluted in sterile PBS to the specified dosing in a volume of 0.5 mL and delivered via IP injection. Groups of 10 mice (~20 g) were administered via IP injection a single dose of negative control pAbs or anti-EBOV pAbs at a dose of 100 mg/kg. Control mice received negative control pAbs 24 hours post challenge, while experimental mice received anti-EBOV pAbs 24 hours or 48 hours post challenge with 100 PFU via IP injection. Mice were observed daily for 28 days from initial challenge for signs of disease. Mouse studies were performed in the BSL-4 laboratory of USAMRIID.

### Statistical analysis

Student’s T-test method was used for analysis of ELISA titers, antibody neutralization titers, and affinity data. For mouse challenge study, a two-tailed Fisher’s exact test was used to analyze mouse survival data on day 28.

## Additional Information

**How to cite this article**: Dye, J. M. *et al.* Production of Potent Fully Human Polyclonal Antibodies against Ebola Zaire Virus in Transchromosomal Cattle. *Sci. Rep.* **6**, 24897; doi: 10.1038/srep24897 (2016).
